# The effects of taxonomy, diet, and ecology on the microbiota of riverine macroinvertebrates

**DOI:** 10.1002/ece3.6993

**Published:** 2020-11-18

**Authors:** Shawn A. Kroetsch, Karen A. Kidd, Wendy A. Monk, Joseph M. Culp, Zacchaeus G. Compson, Scott A. Pavey

**Affiliations:** ^1^ Department of Biological Sciences University of New Brunswick Saint John New Brunswick Canada; ^2^ Canadian Rivers Institute University of New Brunswick Saint John New Brunswick Canada; ^3^ Department of Biology and School of Geography and Earth Sciences McMaster University Hamilton Ontario Canada; ^4^ Environment and Climate Change Canada @ Canadian Rivers Institute Faculty of Forestry and Environmental Management University of New Brunswick Fredericton New Brunswick Canada; ^5^ Environment and Climate Change Canada Department of Biology and Geography and Environmental Studies Wilfrid Laurier University Waterloo Ontario Canada; ^6^ Environment and Climate Change Canada @ Canadian Rivers Institute University of New Brunswick Fredericton New Brunswick Canada; ^7^ Centre for Environmental Genomics Applications (CEGA) St. John’s Newfoundland and Labrador Canada

**Keywords:** bacterial diversity, freshwater aquatic invertebrate, invertebrate‐microbe interactions, microbial ecology, microbiota

## Abstract

Freshwater macroinvertebrates play key ecological roles in riverine food webs, such as the transfer of nutrients to consumers and decomposition of organic matter. Although local habitat quality drives macroinvertebrate diversity and abundance, little is known about their microbiota. In most animals, the microbiota provides benefits, such as increasing the rate at which nutrients are metabolized, facilitating immune system development, and defending against pathogenic attack. Our objectives were to identify the bacteria within aquatic invertebrates and determine whether their composition varied with taxonomy, habitat, diet, and time of sample collection. In 2016 and 2017, we collected 264 aquatic invertebrates from the mainstem Saint John (Wolastoq) River in New Brunswick, Canada, representing 15 orders. We then amplified the V3‐V4 hypervariable region of the 16S rRNA gene within each individual, which revealed nearly 20,000 bacterial operational taxonomic units (OTUs). The microbiota across all aquatic invertebrates were dominated by Proteobacteria (69.25% of the total sequence reads), but they differed significantly in beta diversity, both among host invertebrate taxa (genus‐, family‐, and order‐levels) and temporally. In contrast to previous work, we observed no microbiota differences among functional feeding groups or traditional feeding habits, and neither water velocity nor microhabitat type structured microbiota variability. Our findings suggest that host invertebrate taxonomy was the most important factor in modulating the composition of the microbiota, likely through a combination of vertical and horizontal bacterial transmission, and evolutionary processes. This is one of the most comprehensive studies of freshwater invertebrate microbiota to date, and it underscores the need for future studies of invertebrate microbiota evolution and linkages to environmental bacteria and physico‐chemical conditions.

## Graphical abstract

Freshwater macroinvertebrates play key ecological roles in riverine food webs, though little is known about their microbiota—collections of bacteria that provide several benefits to host organisms. Our objectives were to identify the bacteria within aquatic invertebrates from the mainstem Saint John (Wolastoq) River in New Brunswick, Canada and determine whether their composition varied with taxonomy, habitat, diet, and time of sample collection. Our findings suggest that host invertebrate taxonomy was the most important factor in modulating the composition of the microbiota, likely due to mechanisms of bacterial transmission and evolutionary processes; however, in contrast to previous work, we observed no differences in microbiota among functional feeding groups or traditional feeding habits, and neither water velocity nor microhabitat type structured microbiota variability.

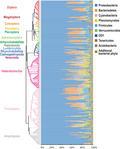

## 1 INTRODUCTION

Aquatic macroinvertebrates are vital members of aquatic ecosystems, driving processes such as nutrient cycling and organic matter breakdown, and aggregating nutrients from the food web base for larger predatory organisms, such as fishes and birds (Wallace & Webster, 1996). Since aquatic invertebrate taxa have differing tolerances to various contaminants, they are also commonly used as indicator species in monitoring the quality of waterways, including the accumulation of metals and insecticides in food webs (Dahl et al., 2004; Luoma et al., 2010; Wallace & Webster, 1996). As with most living animals, aquatic invertebrates possess several communities of bacterial microorganisms that inhabit their body spaces: this is commonly referred to as a microbiota (Lederberg & McCray, 2001; Thursby & Juge, 2017). Together, these bacteria—ranging from commensal to mutualistic to pathogenic—provide beneficial services, including increasing the rate at which nutrients are metabolized (Engel & Moran, 2013), developing immune responses (Ryu et al., 2008; Tang et al., 2012), providing defense from pathogenic attack (Dillon & Dillon, 2004; Osborne et al., 2009; Teixeira et al., 2008), and breaking down recalcitrant food components that would otherwise be indigestible by a host organism (Dillon & Dillon, 2004; Engel & Moran, 2013; Jones et al., 2013; Osborne et al., 2009; Ryu et al., 2008; Tang et al., 2012; Teixeira et al., 2008).

Currently, microbiota studies involving invertebrates have been largely limited to terrestrial species with economic or agricultural value, such as bumble bees and honey bees, which play a vital role in crop pollination (Engel et al., 2012; Martinson et al., 2011, 2012); mosquitoes, which are known vectors of viruses and pathogens (Muturi et al., 2017, 2018); and termites and red palm weevils, which are known herbivorous pests (Ayayee et al., 2015; Kitade, 2004; Mikaelyan et al., 2015; Tagliavia et al., 2014). Overall, these studies have revealed that microbiota are shaped by host taxonomy (Colman et al., 2012; Jones et al., 2013; Mikaelyan et al., 2015; Muturi et al., 2017; Singhal et al., 2017; Yun et al., 2014), environmental changes (Yun et al., 2014), and contaminants (Pennington et al., 2017), and the health of the host is often affected when these bacteria become severely disrupted, as is seen in dysbiosis (Clark & Walker, 2018; Hamdi et al., 2011; Raymann & Moran, 2018). Specifically, studies targeting host taxonomy have found that distinct bacterial taxa are exclusively limited to certain terrestrial invertebrate genera belonging to orders such as Blattodea (cockroaches and termites) (Colman et al., 2012; Kakumanu et al., 2018; Sabree et al., 2012; Sabree & Moran, 2014) and Hymenoptera (ants, bees, and wasps) (Colman et al., 2012; Koch et al., 2013; Kwong & Moran, 2015; Sauers & Sadd, 2019); measures of alpha diversity such as bacterial richness and evenness were also shown to differ significantly among eight invertebrate orders, with alpha diversity measures showing greater similarity among closely related invertebrates (Jones et al., 2013). A previous study comparing microbiota across varying habitats showed significant differences in the relative abundance of anaerobic bacteria, but not in aerobic or facultative anaerobic bacteria (Yun et al., 2014). One study investigating the effects of various contaminants on the Lepidopteran diet found that the bacterial composition changed substantially in the presence of contaminants, as differences in the relative abundance of several operational taxonomic units (OTUs) were observed (Pennington et al., 2017). Finally, dysbiosis of the microbiota in adult honey bees, largely as a result of treatments with antibiotics, has been shown to increase their susceptibility to pathogenic or parasitic infection (Raymann & Moran, 2018).

There were several motivations for conducting this study. Compared with the relatively large focus placed on terrestrial invertebrate microbiota, very little is known of freshwater invertebrate microbiota. To date, only three studies have investigated the microbiota of freshwater stream invertebrates. Those studies have largely focused on determining the impacts of invertebrate functional feeding group and taxonomy on the microbiota (Ayayee et al., 2018; Pechal & Benbow, 2016; Receveur et al., 2020). One study found that the microbiotaof aquatic insects sampled in freshwater streams containing salmon carcasses differed significantly from insects sampled in streams lacking salmon carcasses, leading to the suggestion that the internal bacterial communities of these insects differ as a result of their development and use of the salmon carcasses as a food resource (Pechal & Benbow, 2016). Another study examined ten invertebrate families and sought to determine whether the microbiota of those individuals differed among several functional feeding groups, finding that measures of both alpha and beta diversity differed significantly among functional feeding groups across the invertebrates and that several of these differences were present across two separate streams (Ayayee et al., 2018). Finally, a recent study evaluated the microbiota of several aquatic insect species belonging to different functional feeding groups within an alpine river, demonstrating that functional differences existed between the microbiota of insects from different species and feeding behaviors (Receveur et al., 2020). However, these studies suffer from a notable methodological omission: their analyses of diet or functional feeding group did not control for host invertebrate taxonomy. Consequently, taxonomy may have confounded the overall conclusions that were made regarding the effects of functional feeding group; thus, much of the variation among invertebrate taxa could have been incorrectly attributed to the significant differences found among functional feeding groups. It should be noted that the current study also features some limitations, which are expanded upon in the Discussion.

In this study, we hypothesize that freshwater aquatic invertebrates feature microbiota that are driven by host taxonomic identity as well as dietary and local ecological factors. Due to the limited number of previous studies investigating how several taxonomic, habitat, dietary, or temporal factors shape the composition of the microbiota in aquatic invertebrates, there is a need to both identify which bacterial taxa inhabit aquatic invertebrates, and to understand the natural variability and diversity present within the microbiota of aquatic invertebrates. In the current study, we (a) provide a detailed breakdown of the bacterial taxa present within the microbiota of a diverse set of aquatic invertebrates, (b) examine how host invertebrate taxonomy, water velocity, microhabitat, functional feeding group, traditional feeding habits, and sampling year shape the relative abundance, alpha diversity, and beta diversity of aquatic invertebrate microbiota, and (c) explore how these factors influence the structure of these host‐associated communities within the Saint John (Wolastoq) River (SJWR) in New Brunswick (NB), Canada. As mentioned previously, host taxonomy is the factor which has received the greatest attention to date in terrestrial and aquatic invertebrate microbiota studies (Ayayee et al., 2018; Colman et al., 2012; Jones et al., 2013; Mikaelyan et al., 2015; Muturi et al., 2017; Pechal & Benbow, 2016; Pérez‐Cobas et al., 2015; Receveur et al., 2020; Yun et al., 2014), making it a logical factor to investigate in the current study. The factor of water velocity has not yet been investigated in the context of invertebrate microbiota; previous studies of biofilms have shown that increased water velocities lead to decreased bacterial densities (Soini et al., 2002), raising a question as to whether such differences may impact the invertebrate microbiota. Additionally, environmental bacteria have shown differential growth across aquatic substrates (Goldfarb et al., 2011); thus, we are seeking to understand whether similar patterns exist within invertebrate microbiota sampled across different microhabitats within a sampling site. Given that previous studies of both terrestrial and aquatic invertebrates have revealed significant differences among diets or functional feeding groups (Ayayee et al., 2018; Colman et al., 2012; Jones et al., 2013; Kim et al., 2017; Knapp et al., 2009; Mikaelyan et al., 2015; Pechal & Benbow, 2016; Receveur et al., 2020; Xiang et al., 2019; Yun et al., 2014), this is a factor that will be investigated in the current study. Additionally, the methodological omission noted in previous aquatic invertebrate studies provides increased motivation. Finally, temporal variation has been found to cause significant differences in the bacterial composition of free‐floating bacterioplankton (Portillo et al., 2012) and biofilms (Olapade & Leff, 2005); thus, we are seeking to understand whether temporal variation has any impact on the microbiota of aquatic invertebrates in the current study. This is one of the most comprehensive freshwater invertebrate microbiota studies to date, spanning several ecological factors and invertebrate taxa not previously explored, providing valuable baseline information that further advances the field of environmental microbiology.

## 2 MATERIALS AND METHODS

### 2.1 Field sampling

**Figure 1:**
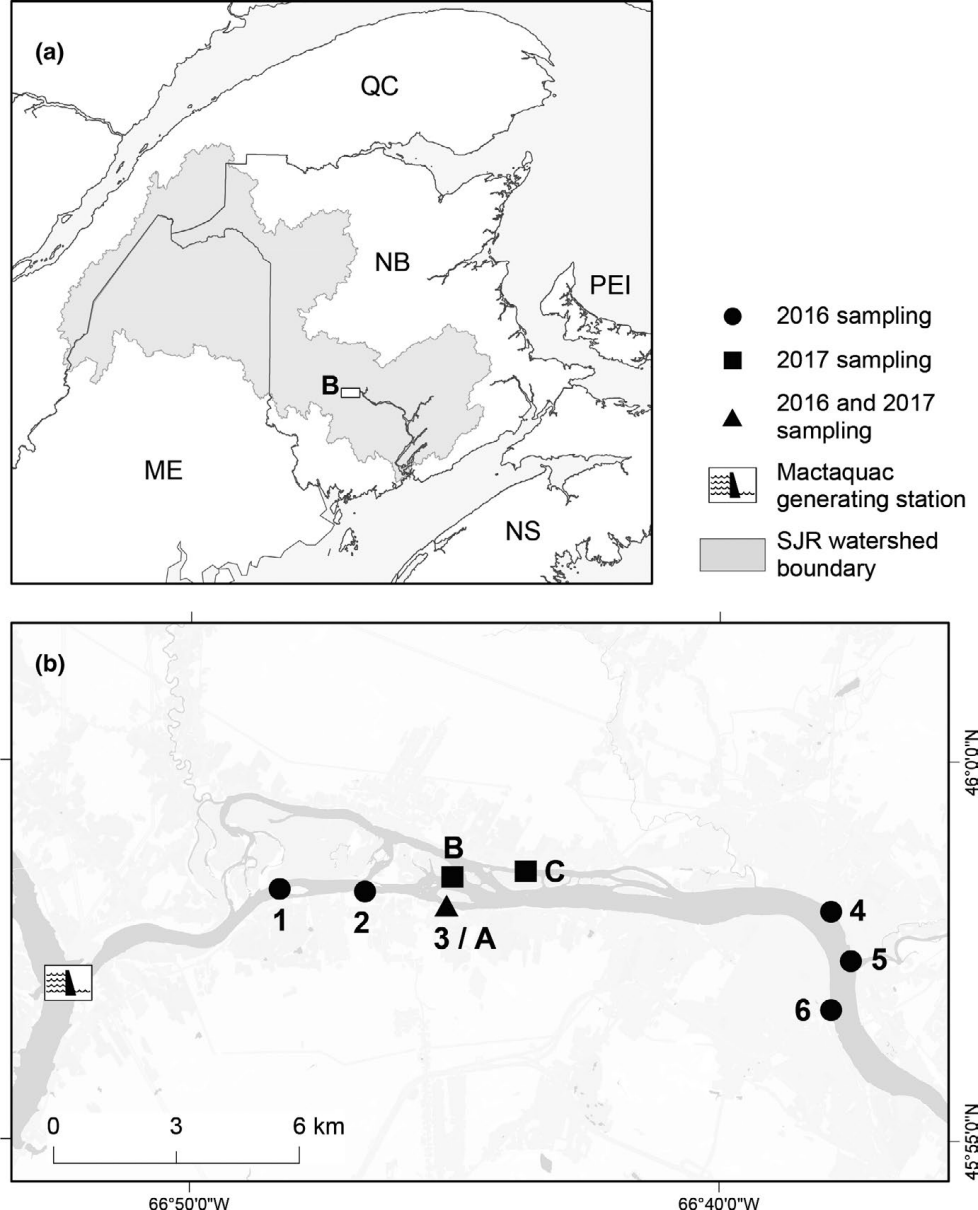
Panel (a) shows the location of the SJWR in New Brunswick, Canada as well as the watershed boundary of the river. The abbreviations in Panel (a) depict the following Canadian provinces: Québec (QC), Prince Edward Island (PEI), Nova Scotia (NS), and New Brunswick (NB), as well as the American state of Maine (ME). Panel (b) shows the 6 sampling sites (labeled 1–6) from 2016 and the 3 sampling sites (labeled A–C) from 2017. Site 3/A was sampled in both 2016 and 2017. All sites are located downstream of the Mactaquac Dam

The SJWR, located primarily in NB, Canada, is 673 kilometers long, with a drainage area of 54,986 square kilometers (Kidd et al., 2011). This river features the Mactaquac Dam, a large, 372 MW run‐of‐the‐river hydroelectric facility in the lower SJWR (Chateauvert et al., 2015). In 2016, six sites along a 20‐kilometer reach of the SJWR were selected (Figure 1) based on previous measurements: sites 1–3 had high‐water velocities (ten measurements recorded for each site with a mean across all three sites of 0.394 ± 0.341 m/s), while sites 4–6, which were located further downstream, had lower velocities (ten measurements recorded for each site with a mean across all three sites of 0.013 ± 0 m/s). Aquatic invertebrate sampling took place from 18 October to 20 October 2016. A kick net (mesh size: 400 µm) was used to collect invertebrates belonging to several taxa and functional feeding groups (and traditional feeding habits) from each of the six sites (Dataset S1). Sites 1–3 (high‐velocity) generally contained more cobble and gravel in the substrate, while sites 4–6 (low‐velocity) generally consisted of a mixture of macrophytes and silt/sand. In 2017, aquatic macroinvertebrates were sampled from three distinct microhabitats (1: cobble/gravel, 2: macrophytes, and 3: silt/sand) within three sampling sites (including site 3/A from 2016) in the SJWR on August 30–31 (Figure 1; Dataset S1). Snapshot measurements of water chemistry were taken from each of the microhabitats using a calibrated YSI Multi‐Meter (ProDSS model, Xylem Inc., Yellow Springs, Ohio): water temperature (°C), dissolved oxygen (% and mg/L), specific conductance (mS/cm), pH, and turbidity (Nephelometric Turbidity Units—NTU) (Table S1). In both years, macroinvertebrates were processed shortly after collection and each live invertebrate was rinsed with 95% ethanol (nondenatured ethyl alcohol) and placed into individual 1.5 ml microcentrifuge tubes filled with 95% ethanol. Tubes were stored in a cooler of dry ice for 3–10 hr until put into a −20°C freezer.

### 2.2 Laboratory sample processing

Prior to DNA extraction, all pipettes and equipment were cleaned with RNase AWAY® Decontamination Reagent (Molecular BioProducts™, Mexico). Using sterile techniques, each invertebrate was first removed from its microcentrifuge tube and surface rinsed with 95% ethanol, followed by a rinse with distilled water to remove excess ethanol. These rinse steps were done to remove environmental bacterial cells present on the exterior surface of invertebrates (Hammer et al., 2015), as only bacterial cells within the individual were of interest in this study. It should be noted that independent tests were not performed to verify the successful removal of environmental bacteria from the samples; however, previous reports indicate that the high bacterial biomass within the invertebrate microbiota often masks the detection of residual environmental bacteria following surface rinsing (Hammer et al., 2015). Whole invertebrates were then individually homogenized using a Retsch™ MM 400 Mixer Mill and both bacterial and invertebrate host DNA were extracted from each sample using the Omega Bio‐tek E.Z.N.A.® Soil DNA Kit. Soil‐specific DNA extraction kits are common in past invertebrate microbiota works, providing suitable concentrations of bacterial DNA for subsequent sequencing applications (Ayayee et al., 2018; Jones et al., 2013; Kim et al., 2017; Knapp et al., 2009; Portillo et al., 2012; Reid et al., 2011).

### 2.3 Aquatic invertebrate barcoding

Extracted macroinvertebrate DNA was amplified using the mitochondrial cytochrome c oxidase subunit I (COI) genetic barcode. A PCR was done using the primer pair LCO1490 (5’‐GGTCAACAAATCATAAAGATATTGG‐3’) and HCO2198 (5’‐TAAACTTCAGGGTGACCAAAAAATCA‐3’), designed specifically for use in identifying invertebrate organisms (Folmer et al., 1994). This particular reaction involved an initial hot‐start step of 95°C for 2 min, followed by 35 cycles of 95°C for 1 min, 40°C for 1 min, and 72°C for 1.5 min; the reaction was finalized with an extension step at 72°C for 7 min (Folmer et al., 1994). This produced a product with a length of 710 bp. Sanger sequencing of these aquatic invertebrate products was completed by the Genome Québec Innovation Centre at McGill University in Montreal, Québec, Canada, using an Applied Biosystems 3730XL DNA Analyzer.

### 2.4 Bacterial metabarcoding

The composition of the bacterial DNA present in the microbiota of each aquatic macroinvertebrate was determined using the 16S rRNA gene. Specifically, the V3‐V4 hypervariable region of 16S rRNA was targeted, as suggested by Illumina©. To amplify this target region, modified versions of the primer pair Bakt_341F (5’‐ACACTGACGACATGGTTCTACACCTACGGGNGGCWGCAG‐3’) and Bakt_805R (5’‐TACGGTAGCAGAGACTTGGTCTGACTACHVGGGTATCTAATCC‐3’), containing tags necessary for Illumina© MiSeq protocols, were used in a PCR (Herlemann et al., 2011). This reaction involved an initial hot‐start step of 95°C for 2 min, followed by 30 cycles of 95°C for 30 s, 62.8°C for 30 s, and 72°C for 1 min; the reaction was finalized with an extension step at 72°C for 8 min, producing sequences of 464 bp. These products were sent to Genome Québec for Illumina© MiSeq PE 300 high‐throughput sequencing. It is important to note that negative (blank) PCR controls were run alongside the bacterial samples to verify that bacterial contaminants were not present, as none of them produced a product. These control samples were not sequenced alongside the bacterial samples, however, to prevent decreases in sampling depth of the bacterial samples, as has been discussed previously (Sampson et al., 2011).

### 2.5 Data analyses

Invertebrate Sanger sequence trimming and analyses were carried out using MEGA7 software (Kumar et al., 2016). Low‐quality base calls, including peaks with uneven spacing and height or that were ambiguous in nature, were manually trimmed from both ends of each sequence trace file, leaving a high‐quality sequence for identification. The BLAST algorithm was used in GenBank (searches employed the standard nucleotide collection database using Megablast optimization for highly similar sequences and a default match/mismatch score of 1,‐2 with linear gap costs) to taxonomically assign the 264 aquatic invertebrates to the levels of genus and species. Upon taxonomic identification of each sample, the invertebrates were assigned to functional feeding groups and traditional feeding habits (Merritt et al., 2008).

Bacterial Illumina© sequence reads were also processed for quality. Low‐quality sequences shorter than 200 bp or possessing a Phred quality score lower than 30 over a 50‐bp sliding window were removed using Trimmomatic v0.38 (Bolger et al., 2014). Unless otherwise stated, all subsequent sequence processing and statistical analyses were completed using QIIME v1.9.1 (Caporaso, Kuczynski, et al., 2010, 2010), R v3.5.1 (R Core Team, 2018), and R Studio v1.1.456 (RStudio Team, 2016). The resulting high‐quality sequences were aligned to the Greengenes core reference alignment using the default PyNAST aligner method in QIIME, and a phylogenetic tree was constructed based on the neighbor‐joining algorithm using MEGA7 (Caporaso, et al., 2010; DeSantis et al., 2006; Kumar et al., 2016). Additionally, the Greengenes bacterial database (v13_8) used in this study's analyses contains over 84 phyla, comprised of previously cultured bacteria and candidate phyla discovered only though culture‐independent metagenomic work (Youssef et al., 2015). OTUs were generated de novo using the default uclust method (Edgar, 2010) in QIIME at a 97% sequence similarity. Chimeric sequences and singletons were filtered out from the remaining high‐quality sequences using the default ChimeraSlayer method (Haas et al., 2011) in QIIME. Taxonomic assignment of OTUs was done using the Greengenes core reference alignment in conjunction with the default uclust method in QIIME (DeSantis et al., 2006; Edgar, 2010; McDonald et al., 2012; Werner et al., 2012). Sequences corresponding to gene fragments from Archaea, chloroplasts, mitochondria, or eukaryotic organisms—possibly resulting from undigested food particles within aquatic invertebrates or from the tissues of the host aquatic invertebrates themselves—were removed following taxonomic identification.

Following sequence processing, remaining high‐quality sequence reads from all invertebrate samples underwent rarefaction to a sequencing depth of 10,000 sequence reads. Rarefaction curves confirmed achievement of adequate sampling depth across samples, indicating that 10,000 sequence reads were sufficient to capture a majority of the diversity within the invertebrate microbiota. Good's coverage index indicated that this sequencing depth covered at least 95% of sequences per sample.

### 2.6 Univariate analyses

In this study, we examined a wide range of aquatic invertebrate taxa and sought to understand the impact of several factors on their microbiota. To better understand whether covariates confounded the results, a univariate approach was taken in which independent “tests groups” were formed to assess how each factor individually impacted the composition of the microbiota. Each univariate test group consisted of individuals that shared the same values for all but one factor, limiting the variation observed in the microbiota to one specific factor—an approach not yet employed in previous invertebrate microbiota literature. The factors being assessed in relation to bacterial microbiota diversity included host invertebrate taxonomy (family‐ and order‐level), habitat (water velocity and microhabitat type), diet (functional feeding groups and traditional feeding habits; only Trichoptera were used for these dietary analyses due to sample size limitations of other invertebrate taxa), and sampling year (see Table 1 for more detail). Multiple comparisons were accounted for following the results of all univariate analyses (calculating differences in relative abundance, alpha diversity, and beta diversity) using the False Discovery Rate procedure (Benjamini, 2010; Benjamini & Hochberg, 1995) with α = 5%.

**Table 1:** List of the individuals and sample sizes included in each of the univariate test groups used to separately evaluate the impacts of host invertebrate taxonomy (family‐ and order‐level), habitat (water velocity and microhabitat type), functional feeding group, traditional feeding habits, and sampling year on the microbiota of aquatic invertebrates

Factor	Univariate test group	Sample size (*n*)	Sample size breakdown per category	Similarities among individuals
Taxonomy (family)	1	18	4 families – 3 Goeridae versus 4 Lepidostomatidae versus 11 Leptoceridae	Collected from site 3/A in 2017
	2	15	3 families – 3 Hydropsychidae versus 7 Lepidostomatidae versus 5 Leptoceridae	Collected from site B in 2017
Taxonomy (order)	1	31	4 orders – 4 Coleoptera versus 3 Diptera versus 3 Ephemeroptera versus 21 Trichoptera	Collected from site 3/A in 2017
	2	30	4 orders – 8 Coleoptera versus 5 Diptera versus 3 Ephemeroptera versus 14 Trichoptera	Collected from site C in 2017
	3	17	3 orders – 4 Ephemeroptera versus 4 Plecoptera versus 9 Trichoptera	Collected from site 2 in 2016, same water velocity
	4	9	3 orders – 3 Diptera versus 3 Hemiptera versus 3 Megaloptera	Collected from site 5 in 2016, same water velocity
Water velocity	1	8	5 low‐velocity versus 3 high‐velocity	Genus *Sialis*, same functional feeding group, collected in 2016
	2	15	11 low‐velocity versus 4 high‐velocity	Family Chironomidae, same functional feeding group, collected in 2016
Microhabitat type	1	30	9 cobble/gravel versus 10 macrophyte versus 11 silt/sand	Genus *Gammarus*, same functional feeding group, collected in 2017
	2	30	7 cobble/gravel versus 12 macrophyte versus 11 silt/sand	Genus *Physella*, same functional feeding group, collected in 2017
	3	17	10 macrophyte versus 7 silt/sand	Genus *Ladislavella*, same functional feeding group, collected in 2017
	4	13	3 cobble/gravel versus 10 silt/sand	Genus *Nectopsyche*, same functional feeding group, collected in 2017
Functional feeding group	1	22	5 collectors versus 5 predators versus 4 scrapers versus 8 shredders	Order Trichoptera, collected from site 3/A in 2017, same microhabitat type
	2	15	3 collectors versus 12 shredders	Order Trichoptera, collected from site B in 2017, same microhabitat type
Traditional feeding habits	1	22	5 carnivores versus 5 herbivores versus 12 omnivores	Order Trichoptera, collected from site 3/A in 2017, same microhabitat type
Sampling year	1	15	3 2016 versus 12 2017	Genus *Physella*, same functional feeding group, collected from site 3/A in 2016 and 2017
	2	8	5 2016 versus 3 2017	Order Ephemeroptera, same functional feeding group, collected from site 3/A in 2016 and 2017

Relative abundances of bacterial sequences observed within the microbiota of all aquatic invertebrates were compared using the group_significance command in QIIME (Caporaso, Kuczynski, et al., 2010, 2010). Nonparametric Kruskal–Wallis tests were run to determine whether the relative abundances of each bacterial OTU differed significantly among various categories of invertebrates being compared for each factor.

Alpha diversity, using both the number of observed OTUs within the microbiota (observed OTU metric) and the Shannon diversity index (*H’*), was assessed for all test groups to estimate how the diversity in the microbiota of individual aquatic invertebrates was impacted by the various factors. Mean alpha diversity values were calculated for samples corresponding to a) host invertebrate taxonomy (family‐ and order‐level), b) water velocity (low‐velocity and high‐velocity), c) microhabitat type (cobble/gravel, macrophyte, and silt/sand), d) functional feeding group (collectors, parasites, piercers, predators, scrapers, and shredders), e) traditional feeding habits (carnivores, herbivores, and omnivores), and f) sampling year (2016 and 2017). Kruskal–Wallis tests were run within the R environment to analyze differences among groups of samples for each of the alpha diversity metrics (R Core Team, 2018). Dunn's test of multiple comparisons using rank sums was run post hoc using the dunn.test package within the R environment to determine which specific pairs of sample groups differed significantly from one another (Dinno, 2017).

Beta diversity was assessed within test groups to determine how the bacterial community dissimilarity differed among individual invertebrates for various factors (i.e., host taxonomy, water velocity, microhabitat type, functional feeding group, traditional feeding habits, and sampling year, as described above). Three metrics were used to assess beta diversity among invertebrate samples: Bray–Curtis dissimilarity, and both unweighted and weighted UniFrac distances. Significant differences in beta diversity, as well as the effect size (*R^2^*), for all test groups, were determined using the Adonis statistical test in QIIME with 999 permutations (Caporaso, Kuczynski, et al., 2010, 2010). Principal Coordinates Analysis (PCoA) plots were generated using the ape package within the R environment to visualize the clustering patterns present within the dissimilarity matrices generated from the bacterial communities among aquatic macroinvertebrates belonging to the various groups of factors (Paradis et al., 2004).

Finally, an additional more stringent filtering protocol was run in parallel with the previously described univariate OTU generation, to better evaluate whether very rare OTUs are responsible for driving the significant differences observed in bacterial relative abundance, alpha diversity, and beta diversity among the microbiota of the invertebrates from this study. Specifically, all OTUs not present in at least 5 invertebrate samples from each univariate test group were filtered out to limit the presence of rare OTUs in the microbial data and corresponding analyses. It should be noted, however, that while this strict filtering resulted in very subtle changes to the findings presented, these small differences ultimately did not change our inferences or alter the overall conclusions made in this study.

### 2.7 Multivariate analyses

A multivariate approach was also used in this study to better control variation through the inclusion of all factors. Specifically, invertebrate samples were organized into two groups by year (78 samples from 2016; 186 samples from 2017) to evaluate the effects of host invertebrate taxonomy at the genus‐, family‐, and order‐level on microbial beta diversity. Using OTUs generated de novo with the uclust method in QIIME at a 97% sequence similarity, OTU tables were created for each sampling year using the make_otu_table command (Caporaso, Kuczynski, et al., 2010, 2010). OTU data within these tables were then converted into presence/absence and relative abundance (values normalized between 0 and 1) OTU tables for both 2016 and 2017. Dissimilarity matrices were subsequently generated with either the Bray–Curtis (for relative abundance OTU tables) or Sorensen (a binary version of Bray–Curtis [for presence/absence‐based OTU tables]) distance metrics using the vegan package within the R environment (Oksanen et al., 2019). Distance‐based redundancy analyses were run on each dissimilarity matrix using vegan to determine the proportion of variance in the microbial data explained by invertebrate taxa at the genus‐, family‐, and order‐levels (Oksanen et al., 2019). Multivariate homogeneity of group dispersions was calculated on each dissimilarity matrix, followed by a permutation test (999 permutations run) of these dispersions using vegan. When significant differences in the microbiota were observed among invertebrate taxa with the multivariate approach, post hoc Tukey's Honest Significant Differences tests were run whereby significant dissimilarities among pairwise taxa were calculated with vegan.

Finally, discriminant function analyses were run for both the 2016 and 2017 OTU datasets using the MASS package within the R environment (Venables & Ripley, 2002). This was done to determine how well invertebrate microbiota communities could predict an invertebrate's assignment to a particular dietary grouping using both the functional feeding group and traditional feeding habit categorizations, following a jackknifing approach. Additional discriminant function analyses were also run to assess how the microbial data of aquatic invertebrates could predict assignment to the water velocity level (2016) or microhabitat (2017) from which an individual was sampled.

## 3 RESULTS

### 3.1 Analysis of invertebrate and bacterial sequences

**Figure 2:**
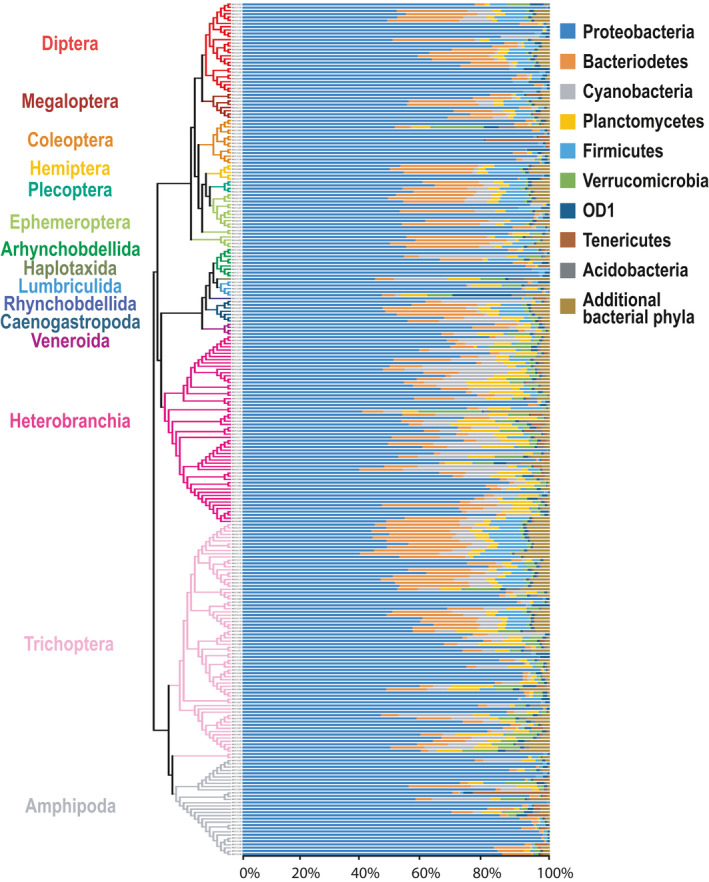
This figure displays both a phylogenetic tree (left) showing the relatedness among each of the 264 aquatic invertebrate samples, and a bar graph (right) summarizing the relative abundances of the most common bacterial phyla found within the microbiota of each aquatic macroinvertebrate sample. The 15 invertebrate orders in the neighbor‐joining phylogenetic tree are represented by colored branches. The composition of the microbiota at the phylum level, using 16S rRNA bacterial sequences from the V3‐V4 hypervariable region, is shown in the bar graph

The V3‐V4 hypervariable region of the 16S rRNA genes associated with the microbiota of 264 aquatic macroinvertebrates from 2016 and 2017 was sequenced and the individuals represented 15 orders, 30 families, and 41 genera (Figure 2; Dataset S1). A total of 23,445,019 16S rRNA gene sequences were obtained from the samples and they clustered into 19,986 unique bacterial OTUs, as defined at a 97% sequence similarity level (Table S2; Dataset S2).

### 3.2 Taxonomic classification of invertebrate microbiota

We documented 48 bacterial phyla, 117 classes, 199 orders, 238 families, and 281 genera within the microbiota of these aquatic macroinvertebrates (Dataset S1). On average, these samples were largely dominated by a small number of bacterial phyla: 87.82% of the bacterial sequences were accounted for by only five phyla; Proteobacteria accounted for 69.25% of the detected sequences, while Bacteroidetes, Cyanobacteria, Planctomycetes, and Firmicutes accounted for 7.67%, 4.86%, 3.19%, and 2.85%, respectively. Three classes of bacteria from the phylum Proteobacteria were dominant within the microbiota of these invertebrates and represented 67.35% of the total bacterial abundance: Gammaproteobacteria (55.95%), Betaproteobacteria (6.26%), and Alphaproteobacteria (5.14%). At the bacterial order level, Enterobacteriales alone represented 54.26% of the detected sequences within the microbiota, with the orders Bacteroidales, Burkenholderiales, Clostridiales, and Sphingomonadales accounting for 4.70%, 3.96%, 1.87%, and 1.42%, respectively. The bacterial family *Enterobacteriaceae* represented 54.26% of the observed sequences within the microbiota of these invertebrates, with the families *Comamonadaceae*, *Pirellulaceae*, *Porphyromonadaceae,* and *Rikenellaceae* representing 2.93%, 1.24%, 1.08%, and 1.06% of the bacterial sequences, respectively. At the genus level, a large percentage of the observed bacterial sequences (an average of 81.88%; Dataset S7) could not be assigned to previously classified bacterial sequences; despite this, the most abundant bacterial genera within these invertebrates were as follows: *Streptococcus* (0.89%), *Delftia* (0.78%), PW3 (0.75%), *Flavobacterium* (0.74%), and *Dysgonomonas* (0.62%). Datasets [Link], [Link], [Link], [Link], [Link] provide complete taxonomic descriptions (at the phylum‐, class‐, order‐, family‐, and genus‐levels) of each bacterial taxon found within the 264 aquatic invertebrates in this study.

### 3.3 Factors influencing relative abundances of bacteria in invertebrate microbiota

When measuring the factors influencing the bacterial relative abundance among invertebrate microbiota, a univariate approach was used. The composition of macroinvertebrate microbiota differed with family‐level taxonomy and sampling year, but less so with dietary habits (Table 2). Specifically, the relative abundances of several bacterial OTUs differed significantly among invertebrate taxa in one of the two univariate test groups for host invertebrate family‐level comparisons (test group 1 for family‐level taxonomy). Similarly, one of the two univariate test groups showed differences in the relative abundances of bacterial OTUs over time (test group 1 for sampling year). Comparisons among functional feeding groups showed differences in less than 1% of all bacterial OTUs (test group 2 for functional feeding group). Finally, the test groups evaluating the impacts of order‐level taxonomy, water velocity, microhabitat type, and traditional feeding habits showed no differences in the bacterial OTU relative abundances.

**Table 2:** Percentage of bacterial OTUs that had significantly different relative abundances among the categories of each factor. Corrections for multiple comparisons were made using the Benjamini–Hochberg procedure and a false discovery rate of 5%

Factor	Univariate test group	% of significantly different bacterial OTUs (number of significantly different OTUs/total number of OTUs)
Taxonomy (family)	1	0%
	2	14.81% (453/3059)
Taxonomy (order)	1	0%
	2	0%
	3	0%
	4	0%
Water velocity	1	0%
	2	0%
Microhabitat type	1	0%
	2	0%
	3	0%
	4	0%
Functional feeding group	1	0%
	2	0.95% (24/2537)
Traditional feeding habits	1	0%
Sampling year	1	11.77% (321/2728)
	2	0%

### 3.4 Factors shaping the alpha diversity of invertebrate microbiota

Taxonomy—but not water velocity, microhabitat, feeding habits, or year—affected the alpha diversity of macroinvertebrate microbiota (Table 3). The family of a host significantly affected the Shannon diversity index (test group 2 for family). Order‐level taxonomy also showed similar results, as test group 2 for order‐level taxonomy showed significant differences in the Shannon diversity index. Finally, the test groups evaluating water velocity, microhabitats, functional feeding groups, traditional feeding habits, and sampling year showed no significant differences in alpha diversity among invertebrates.

**Table 3:** *P* values from Kruskal–Wallis statistical tests measuring alpha diversity in the microbiota of aquatic invertebrates. Corrections for multiple comparisons were made using the Benjamini–Hochberg procedure and a false discovery rate of 5%. *P* values depicting significant differences are bolded

Factor	Univariate test group	Kruskal–Wallis *P* values	
		Observed OTUs	Shannon diversity index (*H'*)
Taxonomy (family)	1	0.432	0.543
	2	0.009	**0.006**
Taxonomy (order)	1	0.094	0.278
	2	0.004	**0.003**
	3	0.146	0.740
	4	0.252	0.066
Water velocity	1	0.655	0.297
	2	0.019	0.009
Microhabitat type	1	0.463	0.026
	2	0.552	0.835
	3	0.079	0.696
	4	0.063	0.043
Functional feeding group	1	0.594	0.098
	2	0.665	0.885
Traditional feeding habits	1	0.430	0.056
Sampling year	1	0.083	0.194
	2	0.025	0.025

### 3.5 Factors shaping the beta diversity of the microbiota

**Figure 3:**
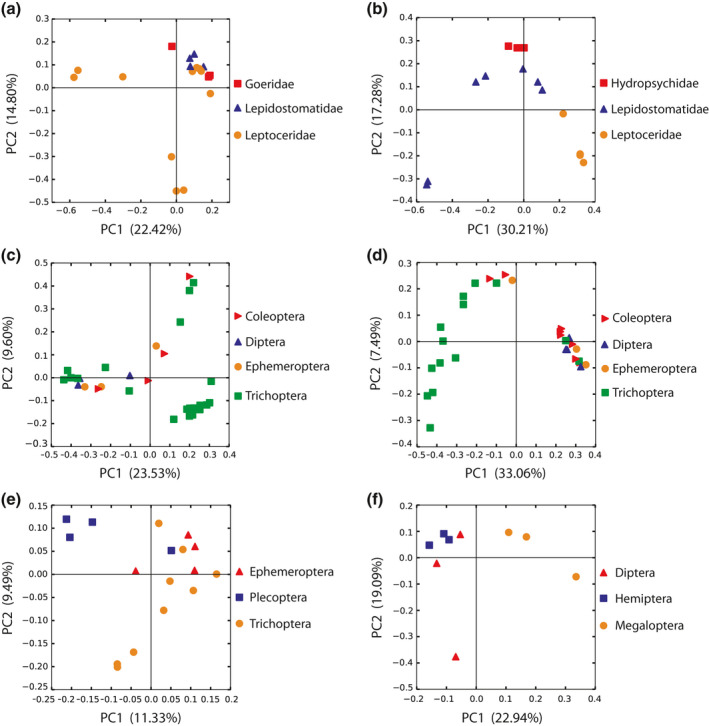
PCoA plots displaying the beta diversity among distinct aquatic invertebrate families (a [test group 1 for family‐level taxonomy] and b [test group 2 for family‐level taxonomy]) and orders (c [test group 1 for order‐level taxonomy], d [test group 2 for order‐level taxonomy], e [test group 3 for order‐level taxonomy] and f [test group 4 for order‐level taxonomy]). The unweighted UniFrac metric was used to construct the dissimilarity matrices from which these plots were generated. Each colored shape represents the microbiota of an individual aquatic invertebrate sample

Several significant differences in the beta diversity of the microbiota of macroinvertebrate taxa were observed using both univariate (Table 4) and multivariate analyses of the data (Table 5). Specifically, univariate analyses revealed differences in beta diversity of microbiota among aquatic invertebrate taxa at the family‐ and order‐levels. Beta diversity differed among invertebrates belonging to different families (1 of 2 univariate groups across all three beta diversity metrics), with effect sizes (*R^2^*) showing that between 12% and 65% of the overall variation in dissimilarities was explained by this level of taxonomy. Similarly, clustering of microbiota among invertebrates from distinct families was observed using a PCoA plot (Figure 3b). Bacterial community structure also differed significantly among orders of macroinvertebrates (4 of 4 univariate groups for unweighted UniFrac); between 11% and 78% of the overall variation in dissimilarities was explained by order‐level taxonomy. The samples evaluating the effects of taxonomy also showed clustering of microbiota among invertebrate orders (Figure 3c–f). Finally, multivariate permutation tests of the group dispersions showed significant differences in beta diversity among microbiota for aquatic invertebrate taxa at the genus‐, family‐, and order‐levels in 2016 and 2017 (Table 5).

**Table 4:** *P* values from Adonis statistical tests measuring beta diversity using the unweighted and weighted UniFrac metrics and the Bray–Curtis dissimilarity metric. Corrections for multiple comparisons were made using the Benjamini–Hochberg procedure and a false discovery rate of 5%. *P* values depicting significant differences in beta diversity are bolded. Effect size (*R* ^2^) values display how much of the overall variation in dissimilarities can be explained by the factor being tested

Factor	Univariate test group	Unweighted UniFrac		Weighted UniFrac		Bray–Curtis dissimilarity	
		*P* values	*R^2^*	*P* values	*R^2^*	*P* values	*R^2^*
Taxonomy (family)	1	0.331	0.121	0.078	0.183	0.193	0.140
	2	**0.001**	0.353	**0.001**	0.645	**0.001**	0.582
Taxonomy (order)	1	**0.014**	0.147	0.182	0.125	0.357	0.105
	2	**0.001**	0.266	**0.014**	0.216	0.077	0.162
	3	**0.002**	0.156	0.026	0.201	0.030	0.168
	4	**0.011**	0.330	**0.005**	0.782	**0.007**	0.725
Water velocity	1	0.215	0.153	0.313	0.137	0.196	0.192
	2	0.025	0.117	**0.002**	0.368	**0.007**	0.317
Microhabitat type	1	0.027	0.101	0.025	0.128	**0.002**	0.152
	2	0.213	0.076	0.616	0.061	0.419	0.069
	3	0.235	0.070	0.224	0.079	0.325	0.063
	4	0.088	0.125	0.064	0.168	0.077	0.154
Functional feeding group	1	0.228	0.160	0.015	0.281	0.051	0.203
	2	0.040	0.129	0.243	0.093	0.247	0.093
Traditional feeding habits	1	0.084	0.130	0.070	0.250	0.130	0.177
Sampling year	1	**0.006**	0.162	**0.003**	0.370	**0.001**	0.288
	2	**0.001**	0.157	**0.006**	0.328	**0.017**	0.250

**Table 5:** *P* values and *F*‐statistics from permutation tests measuring beta diversity using dissimilarity matrices constructed with the Bray–Curtis and Sorensen dissimilarity metrics. *P* values depicting significant differences in beta diversity are bolded

Factor	Sampling Year	Bray–Curtis dissimilarity (relative abundance)		Sorensen dissimilarity (presence/absence)	
		*P* values	*F*‐statistic	*P* values	*F*‐statistic
Taxonomy (genus)	2016	**0.001**	3.558	**0.001**	27.409
	2017	**0.001**	3.905	**0.001**	6.884
Taxonomy (family)	2016	**0.009**	2.546	**0.001**	9.234
	2017	**0.001**	3.950	**0.001**	7.613
Taxonomy (order)	2016	**0.004**	3.683	**0.001**	8.177
	2017	**0.001**	5.841	**0.001**	10.233

**Figure 4:**
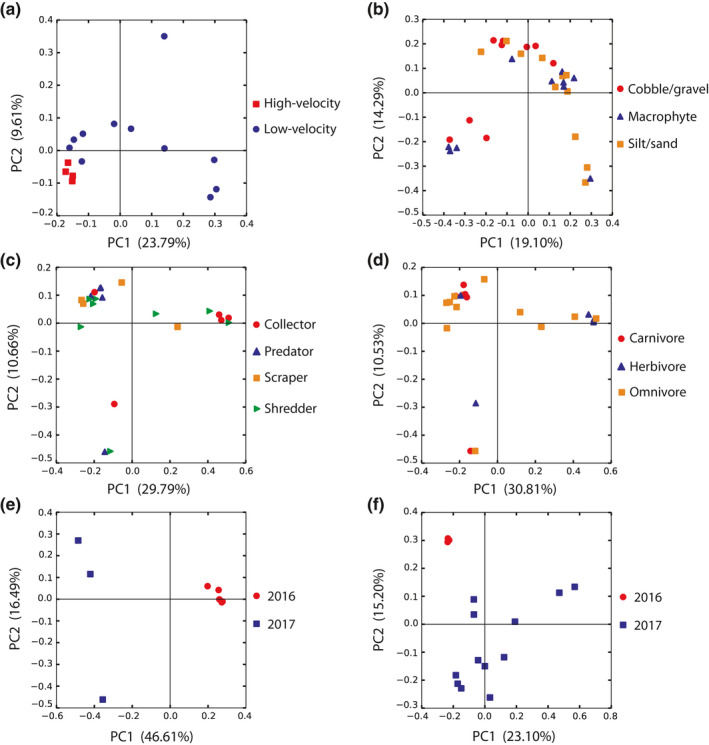
PCoA plots displaying the beta diversity among invertebrates collected from distinct water velocity levels (a [test group 2 for water velocity]), microhabitat types (b [test group 1 for microhabitat type]), functional feeding groups (c [test group 1 for functional feeding group]), traditional feeding habits (d [test group 1 for traditional feeding habits]), and sampling years (e [test group 1 for sampling year] and f [test group 2 for sampling year]). The unweighted UniFrac metric was used to construct the dissimilarity matrices from which these plots were generated. Each colored shape represents the microbiota of an individual aquatic invertebrate sample

Some differences in the beta diversity of invertebrate microbiota among sites varying in water velocities and microhabitats were found using univariate (Table 4) and multivariate approaches. Significant differences in microbiota beta diversity were found among individuals sampled from both low‐water velocity and high‐water velocity sites, with between 12% and 37% of the overall variation attributed to water velocity (one univariate test group for weighted UniFrac and Bray–Curtis dissimilarity). However, overlap was observed among samples from the two water velocity types, suggesting that this habitat characteristic had a weak effect on beta diversity (Figure 4a). Additionally, only one univariate test group showed a significant dissimilarity in microbiota bacterial community structure among samples collected from different microhabitat types, with between 7% and 17% of the overall variation in dissimilarities attributed to this factor. Considerable overlap in microbiota was displayed among samples collected from different microhabitats (Figure 4b). Multivariate linear discriminant function analyses from the 2016 data showed that water velocity was not a strong predictor of an invertebrate's microbial composition. Discriminant functions based on invertebrate microbiota correctly predicted invertebrates sampled from low‐ and high‐water velocity sites at a rate of 48.48% and 53.33%, respectively, which was very close to the 50% null expectation for correct assignment. Multivariate analyses from the 2017 dataset revealed that the sampled microhabitat type was not a strong predictor of the composition of invertebrate microbiota. Specifically, invertebrates sampled from cobble/gravel, macrophyte, and silt/sand microhabitats were correctly assigned back to their microhabitat types using microbial data at a rate of 25.35%, 31.03%, and 29.82%, respectively, which were all below the 33% null expectation for correct assignment (linear discriminant function analyses).

Univariate and multivariate analyses found that both functional feeding groups and traditional feeding habits had little effect on the beta diversity of invertebrate microbiota from the order Trichoptera (Table 4 and 6). Both the two test groups evaluating functional feeding groups and the single univariate test group evaluating traditional feeding habits showed no significant differences in beta diversity. Further, the microbial data from these test groups showed no clustering of the samples according to functional feeding group (Figure 4c) or traditional feeding habits (Figure 4d), as both the 2016 and 2017 data revealed that the functional feeding group of a Trichopteran was not a good predictor of its microbial composition (multivariate discriminant function analyses). Of the four functional feeding groups compared in 2016 (collectors, piercers, predators, and scrapers), invertebrates were correctly assigned to their functional feeding groups, using microbial data, at a rate similar to 25% (the null expectation for correct assignment; discriminant functions using jackknifing; Table 6). Of the three traditional feeding habits compared in 2016, carnivores and herbivores were correctly predicted above the null expectation for correct assignment, while omnivores were predicted at a rate lower than 33% (the null expectation for correct assignment). It should be noted, however, that the carnivores (*n* = 12) and herbivores (*n* = 29) from this comparison had lower sample sizes than the omnivores (*n* = 37), which in part could have contributed to the higher assignment values for the two feeding habits. Similarly, of the six functional feeding groups compared in 2017 (collectors, parasites, piercers, predators, scrapers, and shredders), invertebrates were correctly assigned to their functional feeding groups at rates similar to 17% (the null expectation for correct assignment; discriminant functions using jackknifing). Comparisons among invertebrates with different traditional feeding habits in 2017 revealed correct assignment to an invertebrate's feeding habit at a rate near or below 33% (the null expectation for correct assignment; discriminant functions using jackknifing).

**Table 6:** Percentage of correctly predicted individuals to each functional feeding group and traditional feeding habit following a discriminant function analysis, using a jackknifing approach. Instances where no values appear indicate that no invertebrates were sampled from that functional feeding group during a particular sampling year. “NaN” indicates that no prediction was able to be made due to too small a sample size for invertebrates from the parasite functional feeding group in 2017. The null expectation for correct assignment to the 2016 functional feeding groups was 25% and in 2017 it was 20%, while the null expectation for correct assignment to the traditional feeding habits in 2016 and 2017 was 33%

Sampling year	Functional feeding group					
	Collector	Parasite	Piercer	Predator	Scraper	Shredder
2016	34.09%		33.33%	25.00%	21.05%	
2017	15.63%	NaN	50.00%	21.05%	15.38%	17.91%

Sampling Year	Traditional feeding habits		
	Carnivore	Herbivore	Omnivore
2016	58.33%	44.83%	27.03%
2017	30.00%	35.38%	30.69%

There were significant effects of sampling year on the microbiota of these riverine invertebrates (Table 4). All measures of beta diversity were significantly different between sampling years in both univariate test groups, with between 16% and 37% of the overall variation in dissimilarities attributed to sampling year. Clear patterns of clustering were revealed among aquatic invertebrate samples collected during each sampling year (Figure 4e, f).

## 4 DISCUSSION

In the current study, we characterized the microbiota of 264 aquatic macroinvertebrates from the SJWR in NB, Canada and assessed how the composition of the microbiota of aquatic invertebrates was affected by factors including host taxonomy, measures of habitat, diet, and time. The microbiota of these aquatic invertebrates differed significantly according to host invertebrate taxonomy and sampling year (Dataset S1). In addition, measures of habitat, such as water velocity and microhabitat type, had weak but significant impacts on bacterial composition, while functional feeding group and traditional feeding habits had no significant effect on the microbiota.

When compared to previous microbiota studies of terrestrial invertebrates, the aquatic invertebrates analyzed in this study contained similar bacterial phyla within their microbiota. The bacterial phylum Proteobacteria was dominant within the invertebrates from the SJWR; similarly, terrestrial invertebrates have relative abundances of Proteobacteria that range from 48%–81% (Colman et al., 2012; Jones et al., 2013; Kim et al., 2017; Mikaelyan et al., 2015; Muturi et al., 2017; Pérez‐Cobas et al., 2015; Yun et al., 2014). Several additional bacterial phyla were also common among the freshwater invertebrates from this study: Acidobacteria, Actinobacteria, Bacteroidetes, Cyanobacteria, Firmicutes, Fusobacteria, and Planctomycetes. The bacterial phyla identified in the current study also show similarities to bacteria found in previously studied terrestrial invertebrates, freshwater organisms, and freshwater substrates. A summary of the most common bacterial phyla identified within the microbiota of terrestrial invertebrates (Colman et al., 2012; Hernández‐García et al., 2017; Jones et al., 2013; Mikaelyan et al., 2015; Muturi et al., 2017; Pérez‐Cobas et al., 2015; Rizzi et al., 2013; Singhal et al., 2017; Yun et al., 2014) and freshwater invertebrates (Ayayee et al., 2018; Pechal & Benbow, 2016; Receveur et al., 2020), fishes (Desai et al., 2012; X. Li et al., 2013; Roeselers et al., 2011; Sullam et al., 2012; Wu et al., 2012), plants (Srivastava et al., 2017; Tanaka et al., 2012), sediments (Zhang et al., 2019), bacterioplankton (Ayayee et al., 2018; Portillo et al., 2012), and biofilms (Ayayee et al., 2018) is shown in Table 7. In general, these findings show that the bacterial phyla observed in the microbiota of related freshwater organisms and substrates overlap considerably with the phyla identified in the current study, though there are several bacterial taxa that are more limited to particular organisms and substrates.

**Table 7:** A summary of the most common bacterial phyla found within the microbiota of the freshwater invertebrates from the current study, previous studies of terrestrial invertebrates, and related studies of freshwater invertebrates, fishes, plants, sediments, bacterioplankton, and biofilms

Bacterial phylum	Organisms and substrates							
	Freshwater invertebrates from SJWR	Freshwater invertebrates	Terrestrial invertebrates	Freshwater fishes	Freshwater plants	Freshwater sediments	Freshwater bacterioplankton	Freshwater biofilms
Acidobacteria	✓	✓	✓			✓		
Actinobacteria	✓	✓	✓	✓			✓	
Bacteroidetes	✓	✓	✓	✓			✓	✓
Chloroflexi		✓				✓		
Cyanobacteria	✓	✓	✓					✓
Firmicutes	✓	✓	✓	✓				
Fusobacteria	✓	✓	✓	✓				
Nitrospirae						✓		
Planctomycetes	✓	✓	✓	✓				
Proteobacteria	✓	✓	✓	✓	✓	✓	✓	✓
Verrucomicrobia	✓	✓		✓				

At lower levels of bacterial taxonomy, such as family and genus, considerable taxonomic variability is observed when comparing the results of this study to that of previous terrestrial and aquatic invertebrate works. Despite this, the bacterial family *Enterobacteriaceae*, which dominated the microbiota of the aquatic invertebrates in our study, has been commonly identified in a diverse range of terrestrial and aquatic invertebrates (Ayayee et al., 2018; Colman et al., 2012; Hernández‐García et al., 2017; Muturi et al., 2017, 2018; Pechal & Benbow, 2016; Pérez‐Cobas et al., 2015; Rizzi et al., 2013; Singhal et al., 2017; Yun et al., 2014). *Enterobacteriaceae* includes several opportunistic pathogens and mutualists known to contribute to host nutrition through carbohydrate fermentation, lignocellulose degradation, and nitrogen‐fixation, as well as supporting host development and reproduction (Gurung et al., 2019; Rizzi et al., 2013). Additionally, several bacterial genera including *Burkholderia*, *Flavobacterium*, and *Rickettsia* were commonly abundant across the microbiota of the aquatic invertebrates from this study and have been observed in many previous microbiota studies of terrestrial and aquatic invertebrates (Dataset S7) (Ayayee et al., 2018; Engel & Moran, 2013; Muturi et al., 2017; Pérez‐Cobas et al., 2015; Yun et al., 2014). Species of the genus *Burkholderia* serve as defensive antifungal symbionts, protecting the eggs of their hosts from harmful fungi and microbes in a group of herbivorous beetles (Flórez et al., 2017). Bacteria from the genus *Flavobacterium* have been shown to reduce the reproductive fitness in male ladybird insects (Elnagdy et al., 2014); similarly, members of the genus *Rickettsia* are often pathogenic and have been found to manipulate the reproductive fitness and fertility of host invertebrates (Lawson et al., 2001; Perlman et al., 2006; Sakurai et al., 2005). When comparing the bacterial taxa from this study to those from previous terrestrial and aquatic invertebrate studies, it is important to keep in mind that several ecological factors likely differ among sampling locations (as well as overall geography) that could contribute to some of the observed differences. Additionally, despite the known functions of the above bacteria identified within terrestrial invertebrates, these functions may differ when associated with aquatic invertebrate hosts. Finally, it should be noted that differences in the methods used between the present study and previous studies when collecting, storing, or performing laboratory work may contribute to the observed differences in bacterial abundance and in the composition of the microbiota, as has been suggested previously (Hammer et al., 2015), warranting further investigation.

As previously reported for a wide range of terrestrial and aquatic invertebrates (Colman et al., 2012; Jones et al., 2013; Mikaelyan et al., 2015; Muturi et al., 2017; Receveur et al., 2020; Singhal et al., 2017; Yun et al., 2014), we found significant differences in the relative abundance, alpha diversity, and beta diversity of the microbiota among host aquatic invertebrate taxa at the levels of genus, family, and order. Core microbiota, which are collections of specific bacterial species commonly shared among all individuals of a host invertebrate taxon (Pérez‐Cobas et al., 2015), may be responsible for these observed differences. The processes by which core microbiota develop in invertebrates are not known; however, previous studies of terrestrial invertebrates point to several factors that may contribute to their formation and maintenance. Vertical transmission of bacteria from parent to offspring is supported by studies investigating the effects of taxonomy on the microbiota in cockroaches (Kakumanu et al., 2018; Sabree et al., 2012; Sabree & Moran, 2014), bumble bees (Koch et al., 2013; Kwong & Moran, 2015; Sauers & Sadd, 2019), honey bees (Koch et al., 2013; Kwong & Moran, 2015), and termites (Sabree et al., 2012; Sabree & Moran, 2014). Each of these invertebrates are found to feature specific bacterial species within their core microbiota that are observed consistently across all individuals regardless of diet, suggesting that particular bacteria have been passed down vertically from parent to offspring.

The horizontal transmission of bacteria among individuals of the same generation has also been hypothesized as a reason for the formation and maintenance of core microbiota. Highly social invertebrates, such as wasps, partake in trophallaxis where nestmates exchange regurgitated liquids between one another and, in the process, likely exchange bacteria (Nalepa et al., 2001). Similarly, honey bees, which are also highly social, possess bacterial symbionts within their microbiota that are acquired during the first few days of their adult life stage through social interactions with other adult workers in their colony (Martinson et al., 2012; Powell et al., 2014). In addition to social invertebrates, several taxa are known to partake in coprophagy—the ingestion of feces—which could lead directly to the introduction of specific fecal and environmental bacteria into their microbiota (Nalepa et al., 2001).

In addition to the vertical and horizontal transmission of bacteria, both the microbiota and their hosts are subject to evolutionary forces that drive the composition of the core microbiota. Much of the previous microbiota research has focused on how selective pressures within the microbiota directly affect the host. However, it has been suggested that through a combination of strong selective pressures driving hosts to maintain a beneficial microbiota and through evolutionary competition among bacterial taxa attempting to persist within their invertebrate host, a stable but dynamic bacterial ecosystem is maintained by the host (Gupta & Nair, 2020). This then suggests a pattern of covariation between core microbiota and host taxonomy; related invertebrate taxa experience similar selective pressures and as a result possess similar core microbiota. The concept of coevolution, in which there is a reciprocal and adaptive change in allele frequencies between a bacterial symbiont and its host (Woolhouse et al., 2002), often accompanied by a reduction in the genome of the bacterial partner that can limit their replication to solely within the host (Gupta & Nair, 2020; Moran & Plague, 2004), is also supported within this framework. Coevolution has been observed between the cockroach *Blatetella germanica* and *Blattabacterium* strain Bge, in which the intracellular endosymbiotic bacterium has evolved to play a vital role in nutrient acquisition for the cockroaches, while recycling nitrogenous wastes produced by the host (Pérez‐Cobas et al., 2015). Similarly, the evolutionary divergence of termites from cockroaches resulted in the complete loss of *Blattabacterium* paired with the acquisition of specialized wood‐degrading bacteria in termites (Sabree et al., 2012). Overall, it is likely that both vertical and horizontal factors, combined with one or more of the presented evolutionary processes, play a role in how terrestrial invertebrate core microbiota are colonized and maintained, though further research is necessary to determine whether similar trends exist in aquatic invertebrates.

Previous studies involving freshwater fishes also support the existence of core microbiota. Several core bacterial taxa were commonly shared among zebrafish from the laboratory and those collected from natural habitats (Roeselers et al., 2011). Anadromous salmon have also been shown to feature distinct core microbiota; individuals sampled from both freshwater and saltwater habitats shared common core bacteria despite the differing environmental conditions (Rudi et al., 2018). A study involving freshwater rainbow trout (*Oncorhynchus mykiss*) revealed the presence of *Carnobacterium maltaromaticum* among all sampled individuals; notably, this bacterium was not found in related species of similar dietary groups (Desai et al., 2012). Finally, the core microbiota of freshwater grass carp (*Ctenopharyngodon idellus*) has been identified and described, and is largely composed of cellulose‐decomposing bacteria (Wu et al., 2012).

Although taxonomic effects have been widely reported in previous studies involving terrestrial invertebrates and freshwater fishes, the univariate and multivariate results from our study also suggest that aquatic invertebrates have a core microbiota that varies among taxa. In this study, we have provided a description of the bacterial OTUs that primarily comprise the core microbiota of each invertebrate taxon (family‐ and order‐level) from each univariate test group evaluating taxonomy (Datasets [Link], [Link], [Link], [Link], [Link], [Link]). However, despite the support that the current study provides for the existence of core microbiota, previous studies suggest that core microbiota can vary greatly in composition and are capable of taking on several unique forms (healthy compared to dysbiotic, for example), though the cause of these variations is currently unknown (Engel et al., 2016; Li et al., 2015). Specifically, a study involving bees of the genus *Bombus* revealed two distinct core microbiota (referred to in the paper as bacterial “enterotypes”) across the samples belonging to this genus (Li et al., 2015). One enterotype was dominated by well‐known enterobacterial species, while the other was dominated by bacterial species widely regarded as insect pathogens (Li et al., 2015). The authors note that precisely how these dramatic differences in the microbiota occur is not currently known, and how the health of the host bee is consequently affected by these differences is also uncertain (Li et al., 2015). In the current study, while we have identified the bacterial OTUs most likely to form the core microbiota of each invertebrate taxon, we caution that due to a lack of baseline information known regarding the environmental bacteria found at each sampling site, there is a possibility that some of the identified core bacteria may in fact be transient environmental bacteria rather than resident bacteria native to the microbiota. To alleviate these concerns, we suggest that if core microbiota are to be accurately identified in aquatic invertebrates going forward, a greater emphasis should be placed on collecting and sequencing environmental bacterial samples from the water column in each sampling location where invertebrates are collected. This practice would increase the likelihood of accurately identifying the core microbiota, as differentiation would be possible between transient environmental bacteria and resident core bacteria.

Although no previous studies examined whether temporal variability results in differences to the microbiota of aquatic invertebrates, we found a significant temporal effect on bacterial relative abundance and beta diversity. Bacterial communities in streams vary over time. Specifically, biofilms—which are collections of bacterial organisms that often adhere to surfaces such as rocks, small woody debris, and leaves—can differ in the abundances of Alphaproteobacteria, Betaproteobacteria, and Gammaproteobacteria among seasons (Olapade & Leff, 2005); temporal differences in dissolved organic materials and inorganic nutrients likely drive differences among biofilms (Olapade & Leff, 2005; Samways et al., 2017, 2018). Temporal differences in alpha and beta diversity have also been found in free‐floating communities of bacterioplankton in freshwater streams that appeared to be driven by changes in stream water biogeochemistry (Portillo et al., 2012). Given these previously observed temporal changes, it is possible that the temporal differences in the microbiota of the aquatic invertebrates from our study might also have resulted from shifts in biogeochemical conditions. Further research is needed to determine the role of abiotic factors in explaining differences in aquatic invertebrate microbiota over time. Invertebrate microbiota studies should also report sample collection dates, as these are often missing, to ensure that temporal differences can be assessed and considered when interpreting results and to reflect accurate metadata for future meta‐analyses.

The finding that functional feeding group had no significant impact on the microbiota of these samples is contrary to previous research. Of the few studies that previously explored the microbiota of aquatic invertebrates in freshwater environments, measures of alpha and beta diversity differed significantly among the functional feeding groups of aquatic invertebrates (Ayayee et al., 2018). More specifically, estimates of bacterial richness and evenness were greatest in grazers/collectors and lowest in predators and omnivores (Ayayee et al., 2018), with significant differences in bacterial relative abundance found for several bacterial orders among aquatic invertebrates having different dietary sources (Pechal & Benbow, 2016). Functional feeding groups also clustered separately from one another, with omnivorous invertebrates having the most similar beta diversities among streams (Ayayee et al., 2018), and significant functional differences were revealed among the microbiota of invertebrates differing in feeding behavior (Receveur et al., 2020). Diet has also been found to affect the abundance and diversity of terrestrial invertebrate microbiota (Colman et al., 2012; Jones et al., 2013; Kim et al., 2017; Knapp et al., 2009; Mikaelyan et al., 2015; Xiang et al., 2019; Yun et al., 2014), though inconsistencies in the terminology used to classify dietary guilds among research groups make them somewhat difficult to compare. For example, the use of terminology such as “carnivorous,” “scavengers,” “detritivorous,” “nectarivorous,” “pollenivorous” is generally used in only a few studies (Colman et al., 2012; Jones et al., 2013; Kim et al., 2017; Yun et al., 2014); in contrast, terminology such as “omnivores,” “herbivores,” and “predators” is more commonly shared among studies (Colman et al., 2012; Jones et al., 2013; Xiang et al., 2019; Yun et al., 2014). To address the diverse terminology more commonly used in terrestrial invertebrate microbiota studies, we also grouped the aquatic invertebrates according to the traditional feeding habits “carnivores,” “herbivores,” and “omnivores,” though no significant differences were observed in the bacterial abundance, alpha diversity, or beta diversity across these groupings. Additionally, as mentioned previously, there was a notable omission with the methodology used in the previous aquatic invertebrate functional feeding group studies, as the analyses did not control for host invertebrate taxonomy (Ayayee et al., 2018; Pechal & Benbow, 2016; Receveur et al., 2020), and neither sample sizes nor sampling date and time were reported (Ayayee et al., 2018). Since this issue increases the possibility that taxonomy may confound the overall conclusions regarding the effects of functional feeding group, much of the variation among invertebrate taxa may have been incorrectly attributed to functional feeding group. In the current study, only invertebrates from the order Trichoptera (due to poor sample sizes for comparisons using other invertebrate taxa) were included in each of the two univariate test groups used to determine the effects of functional feeding group and in the single test group evaluating traditional feeding habits, thereby reducing the effects of taxonomy as a confounding factor. Similarly, the multivariate approach also allowed this variation to be controlled through the inclusion of all factors.

Measures of habitat, including both water velocity and microhabitat type, were associated with few significant compositional differences in the microbiota of the aquatic invertebrates from this study. While this has not been investigated previously using riverine invertebrates, changes in habitat are associated with compositional differences in the microbiota of broad‐scale habitats. Specifically, one study found that only the relative abundance of anaerobic bacteria differed among invertebrates sampled from four unique habitat types: anaerobic bacteria were more abundant in invertebrates from “aquatic” and “underground” habitats, while invertebrates from the “sky” and “ground” had the lowest abundance of anaerobes (Yun et al., 2014). It is important to note, however, that differences (such as oxygen availability) among these habitat types were likely much larger than those among the microhabitats in our study. The invertebrate microbiota in the current study may vary across habitats and microhabitats because of local differences in environmental bacteria, which have been found to vary with hydrology and physiochemical conditions (Portillo et al., 2012), pH (Methé & Zehr, 1999), dissolved organic carbon (Judd et al., 2006), and temperature (Adams et al., 2010). In addition, aquatic microbial communities differ based on incubation time (Newman et al., 2015) and display successional patterns (Brasell et al., 2015; Jackson et al., 2001; Veach et al., 2016). Additional measures of water chemistry are recommended in future studies as they may prove to have greater influences on the microbiota of macroinvertebrates.

In this study, we have detailed the composition, abundance, and diversity of the microbiota of 264 individual aquatic invertebrates using culture‐independent methodologies. Most notably, our results support the growing body of literature showing significant differences in the microbiota of invertebrates among host taxa at the genus‐, family‐, and order‐levels. This lends support to the existence of core microbiota between distinct host invertebrate taxa and specific bacteria, though further study is needed to determine the origins of core microbiota and to identify the specific essential bacterial organisms involved. We also observed temporal differences in the microbiota of these aquatic invertebrates, which may be related to changes over time in the physical or chemical environments. Additionally, we found almost no significant differences in either the relative abundance or the diversity of the microbiota among invertebrates belonging to different functional feeding groups or traditional feeding habits, contrary to previous findings (Ayayee et al., 2018; Pechal & Benbow, 2016; Receveur et al., 2020). Finally, some weak but significant differences were revealed in the microbiota of these aquatic invertebrates among water velocities and microhabitat types, though these factors appear to be weaker controls of invertebrate microbiota than those such as water quality, which affects natural bacterial communities. Limitations with this study include: sampling sites that differ in terms of their proximity to more densely populated urban areas; low sample sizes for certain aquatic invertebrate taxa and test groups; the use of a small portion of extracted DNA from each sample to perform PCR amplification and high‐throughput sequencing rather than the entire volume of extracted DNA; the potential for PCR bias among bacterial sequences; homogenizing whole invertebrates which presents the risk of including endosymbionts in the analysis of the microbiota; and not collecting environmental bacterial samples at the sampling sites to establish a bacterial baseline against which to compare the microbiota. Yet, this study establishes a baseline of natural variability and diversity of aquatic invertebrate microbiota, and outlines how several ecological factors impact the microbiota. This study is of value in invertebrate microbiology, as it provides new knowledge regarding the relationships between aquatic macroinvertebrates and their associated bacteria, which could allow for future applications related to water quality monitoring and conservation.

## CONFLICT OF INTEREST

The authors declare no conflicts of interest.

## AUTHOR CONTRIBUTION

**Shawn A Kroetsch:** Conceptualization (equal); Data curation (lead); Formal analysis (lead); Funding acquisition (equal); Investigation (lead); Methodology (lead); Visualization (lead); Writing‐original draft (lead); Writing‐review & editing (lead). **Karen A Kidd:** Conceptualization (equal); Funding acquisition (equal); Project administration (equal); Resources (equal); Supervision (equal); Writing‐original draft (supporting); Writing‐review & editing (supporting). **Wendy A Monk:** Conceptualization (equal); Methodology (supporting); Resources (equal); Software (supporting); Visualization (supporting); Writing‐original draft (supporting); Writing‐review & editing (supporting). **Joseph M Culp:** Conceptualization (equal); Writing‐original draft (supporting); Writing‐review & editing (supporting). **Zacchaeus G Compson:** Conceptualization (equal); Methodology (equal); Writing‐original draft (supporting); Writing‐review & editing (supporting). **Scott A Pavey:** Conceptualization (equal); Funding acquisition (equal); Project administration (equal); Resources (equal); Supervision (equal); Writing‐original draft (supporting); Writing‐review & editing (supporting).

## Supporting information

Table S1

Table S2

Dataset S1

Dataset S2

Dataset S3

Dataset S4

Dataset S5

Dataset S6

Dataset S7

Dataset S8

Dataset S9

Dataset S10

Dataset S11

Dataset S12

Dataset S13

## Data Availability

The COI gene sequences of the aquatic macroinvertebrate samples and the 16S rRNA bacterial gene sequences identified from the microbiota of each aquatic invertebrate reported in this study were submitted to NCBI under the accession numbers MT186286 to MT186549 and SRR10989484 to SRR10989747, respectively (Dataset S1). Additionally, the scripts used to remove low‐quality bacterial Illumina© sequences in Trimmomatic v0.38, to perform univariate bioinformatic analyses in QIIME v1.9.1, and to perform multivariate analyses in R v3.5.1 can be found at the following GitHub repository: https://github.com/skroetsc‐unb/The‐Effects‐of‐Taxonomy‐Diet‐and‐Ecology‐on‐the‐Microbiota‐of‐Riverine‐Macroinvertebrates.
