# Supplementary material for: The effects of taxonomy, diet, and ecology on the microbiota of riverine macroinvertebrates
Source: Ecol Evol. 2020 Nov 18;10(24):14000–19. doi: 10.1002/ece3.6993 (PMC7771166; doi:10.1002/ece3.6993)
Supplement: Supplementary file 1 — Table S1 [file ECE3-10-14000-s001.pdf]

TABLE S1. Raw water chemistry data (water temperature, dissolved oxygen, specific conductance, pH, and turbidity) collected from each microhabitat at all sites sampled in 2017.

Site ID	Microhabitat	Date/Time	Water temperature (°C)	Dissolved oxygen (%)	Dissolved oxygen (mg/L)	Specific conductance (mS/cm)	pH	Turbidity (NTU)
3/A	Cobble/gravel	August 30th, 2017 @ 13:05 ADT	21.7	111.7	9.85	122.6	7.91	2.2
	Macrophytes	August 30th, 2017 @ 13:00 ADT	21.6	111.5	9.83	123.0	7.26	3.0
	Silt/sand	August 30th, 2017 @ 13:10 ADT	21.5	406.8	9.43	118.8	7.99	1.7
B	Cobble/gravel	August 30th, 2017 @ 17:15 ADT	21.3	105.4	9.36	117.4	8.14	1.6
	Macrophytes	August 30th, 2017 @ 17:10 ADT	21.4	112.3	9.93	116.7	7.99	1.8
	Silt/sand	August 30th, 2017 @ 17:20 ADT	21.4	127.9	11.40	116.6	8.90	7.2
C	Cobble/gravel	August 31st, 2017 @ 12:20 ADT	21.7	111.4	9.83	118.8	8.32	1.6
	Macrophytes	August 31st, 2017 @ 12:25 ADT	21.9	113.5	9.94	119.2	8.33	2.3
	Silt/sand	August 31st, 2017 @ 12:30 ADT	20.9	102.2	9.12	118.3	8.19	1.6
