# Supplementary material for: The effects of taxonomy, diet, and ecology on the microbiota of riverine macroinvertebrates
Source: Ecol Evol. 2020 Nov 18;10(24):14000–19. doi: 10.1002/ece3.6993 (PMC7771166; doi:10.1002/ece3.6993)
Supplement: Supplementary file 2 — Table S2 [file ECE3-10-14000-s002.pdf]

TABLE S2. Bacterial genomic metadata from the microbiota of 264 aquatic invertebrates. These data were generated following Illumina© MiSeq PE300 with the bacterial primer pair Bakt_341F/Bakt_805R, and following the steps described in the “Data analyses” portion of the “Materials and Methods” section.

Step of quality filtering	Number of bases/sequence reads/operational taxonomic units (OTUs)
Total bases	25,836,649,800
Raw sequence reads	43,061,083
Sequence reads following size selection and quality filtering	25,751,327
High-quality paired sequence reads	23,445,019
Total OTUs	515,551
Unique OTUs (chimeras and singletons removed)	19,986
